# Hyperspectral Estimation of Layer-Specific Leaf Nitrogen Content in Potato Canopy by Integrating Fractional-Order Derivatives and Three-Band Spectral Indices

**DOI:** 10.3390/plants15132045

**Published:** 2026-07-01

**Authors:** Ming Jin, Liaoyuan Ma, Liang Cheng, Zhiying Liu, Zijun Tang, Wangyang Li, Ruiqi Du, Tao Sun, Youzhen Xiang, Fucang Zhang

**Affiliations:** 1 College of Water Resources and Architectural Engineering, Northwest A&F University, Yangling 712100, China; jinming1120@nwafu.edu.cn (M.J.); 2023012608@nwsuaf.edu.cn (L.M.); chengliang2@nwsuaf.edu.cn (L.C.); 2024055914@nwsuaf.edu.cn (Z.L.); zijuntang@pku.edu.cn (Z.T.); lwy1222@nwsuaf.edu.cn (W.L.); 2021050986@nwsuaf.edu.cn (T.S.); 2 Xinjiang Research Institute of Agriculture in Arid Areas, Urumqi 830091, China; 3 Key Laboratory of Agricultural Soil and Water Engineering in Arid and Semiarid Areas, Ministry of Education, Northwest A&F University, Yangling 712100, China; 4 College of Geomatics, Xi’an University of Science and Technology, Xi’an 710054, China; duiruiqi@126.com; 5 Key Laboratory of Northwest Oasis Water-Saving Agriculture, Ministry of Agriculture and Rural Affairs, Xinjiang Academy of Agricultural and Reclamation Science, Shihezi 832000, China

**Keywords:** potato canopy, leaf nitrogen content, vertical heterogeneity, hyperspectral remote sensing, fractional-order derivative, three-band spectral indices, partial least squares regression

## Abstract

To address the insufficient characterization of vertical heterogeneity in potato canopy leaf nitrogen content (LNC), this study developed a layer-specific LNC estimation framework based on canopy hyperspectral reflectance, fractional-order derivative (FOD) transformation, and two-band and three-band optimized spectral indices. Partial least squares regression (PLSR) was then used to evaluate the predictive ability of the selected spectral indices for Top, Middle, and Bottom LNC. Field experiments were conducted from 2022 to 2023 in the semi-arid region of Yulin, Shaanxi Province, China. Canopy hyperspectral reflectance from 350 to 1830 nm and LNC measurements of upper (Top), middle (Middle), and lower (Bottom) leaves were synchronously acquired during the tuber formation stage. The results showed that potato canopy LNC exhibited a clear vertical gradient, following the order Top LNC > Middle LNC > Bottom LNC. Traditional vegetation indices were significantly correlated with LNC, but their correlations decreased with increasing canopy depth, with the highest correlation for Bottom LNC being only 0.524. Compared with traditional vegetation indices, FOD-based two-band indices showed stronger Pearson correlations with layer-specific LNC. Under FOD1.5, the maximum absolute Pearson correlation coefficients (|r|) between the selected two-band indices and LNC reached 0.855, 0.849, and 0.814 for Top, Middle, and Bottom LNC, respectively. The three-band optimized spectral indices further enhanced spectral information extraction, with maximum |r| values of 0.893, 0.885, and 0.852, respectively. However, cross-year validation produced substantially lower R^2^ values, indicating limited temporal transferability of the selected indices and the need for further validation before broader application. Compared with the traditional vegetation index model, it increased the testing-set R^2^ for Bottom LNC by 0.279 and reduced RMSE from 0.159 to 0.113. These results suggest that FOD1.5-integrated three-band optimized spectral indices can improve the indirect estimation of layer-specific LNC from canopy reflectance, particularly for Bottom LNC, where the reflectance–LNC association is affected by canopy signal attenuation and mixing. The findings provide a methodological reference for describing canopy vertical nitrogen status and functional heterogeneity in potato, while their broader applicability requires further validation across growth stages, cultivars, sites, and nitrogen management conditions.

## 1. Introduction

Potato (*Solanum tuberosum* L.) is an important food and economic crop worldwide, playing a critical role in ensuring food security, increasing farmers’ income, and supporting dryland agricultural production [1]. In the semi-arid region of northwestern China, potato is widely cultivated because of its strong adaptability to cool climates and relatively dry environments. However, this region is generally characterized by limited precipitation, strong evaporation, low soil water- and nutrient-retention capacity, and large spatiotemporal fluctuations in water and nutrient supply, making potato production highly dependent on rational water and fertilizer management. Nitrogen is a key nutrient element affecting canopy establishment, photosynthetic capacity, dry matter accumulation, and tuber formation in potato, and its supply level is directly associated with yield formation and nitrogen use efficiency [1]. Insufficient nitrogen supply can restrict leaf expansion and photosynthesis, thereby inhibiting plant growth, whereas excessive nitrogen application may delay maturity, reduce nitrogen use efficiency, and increase nitrogen loss and environmental risks. Therefore, accurate and rapid diagnosis of potato nitrogen nutritional status is of great importance for optimizing nitrogen fertilizer management and improving production efficiency in semi-arid potato-growing regions.

Leaf nitrogen content (LNC) is a direct indicator of crop nitrogen nutritional status and is closely related to chlorophyll content, photosynthetic enzyme activity, leaf physiological function, and biomass accumulation. As a major component of chlorophyll, photosynthetic proteins, and enzymes, nitrogen affects light absorption, photosynthetic capacity, CO_2_ fixation, and carbon assimilation; therefore, LNC is commonly used to evaluate crop nitrogen supply level and physiological activity [2,3]. Although canopy greenness and soil plant analysis development (SPAD) chlorophyll meter readings provide simple and low-cost optical indicators of leaf chlorophyll status and greenness, they remain indirect proxies for nitrogen status. In contrast, LNC more directly reflects nitrogen accumulation and allocation within plants. However, conventional LNC determination mainly relies on destructive sampling, drying, grinding, digestion, and chemical analysis. Although these methods provide relatively reliable measurements, they are labor-intensive, time-consuming, operationally complex, and difficult to apply for repeated and spatially continuous field monitoring, which limits their application in rapid field diagnosis and precision nitrogen management.

Hyperspectral remote sensing provides an effective technical approach for rapid and non-destructive monitoring of crop nitrogen nutrition. However, hyperspectral reflectance does not directly measure LNC; rather, it serves as an indirect optical proxy related to nitrogen status through spectral responses associated with pigment absorption, red-edge variation, near-infrared structural scattering, and shortwave infrared biochemical absorption. These spectral responses are closely associated with physiological and biochemical parameters such as crop chlorophyll, nitrogen, water content, dry matter, and canopy structure [3,4]. Fu et al. systematically summarized the application of hyperspectral remote sensing in crop nitrogen status monitoring and suggested that hyperspectral technology can be used to indirectly estimate crop nitrogen status and its spatiotemporal variation in a real-time and non-destructive manner [3]. In potato studies, Guo et al. estimated potato canopy nitrogen content using hyperspectral index optimization and showed that optimized spectral indices could improve the accuracy of potato nitrogen monitoring [5]. These studies indicate that hyperspectral remote sensing has considerable potential as an indirect tool for potato nitrogen diagnosis.

However, most existing remote sensing studies on potato nitrogen have focused on whole-plant nitrogen, canopy nitrogen content, or canopy-averaged nitrogen indicators. In these studies, the canopy is usually treated as a relatively homogeneous unit, while the vertical heterogeneity of leaf nitrogen content among different canopy layers has received limited attention. In fact, nitrogen distribution within crop canopies is not uniform. Li et al. reported that non-uniform vertical nitrogen distribution commonly exists within plant canopies and is closely related to light environment, leaf age, canopy structure, and nitrogen remobilization [6]. Ignoring differences in internal canopy nitrogen distribution may reduce the explanatory power and practical value of remote sensing-based nitrogen monitoring models. Potato canopy LNC may vary among Top, Middle, and Bottom leaves because of differences in nitrogen supply, leaf position, light environment, canopy structure, and leaf physiological status [6,7,8,9]. However, whether the proportional relationships of LNC among canopy layers remain stable under different nitrogen fertilizer levels is still unclear. Clarifying this vertical LNC distribution is important for interpreting layer-specific nitrogen status and for evaluating the feasibility of estimating Top, Middle, and Bottom LNC from top-of-canopy hyperspectral reflectance. Since canopy reflectance measured above the canopy represents an integrated mixed signal rather than layer-specific leaf reflectance, the relationship between canopy-scale spectral information and vertical LNC distribution needs to be examined statistically.

For potato, differences among canopy layers may be particularly important. During tuber formation and bulking, the potato canopy gradually becomes closed, and leaves at different canopy layers experience markedly different light conditions, leaf age structures, and physiological functions [6,7,8,9]. Upper leaves usually receive stronger direct radiation and maintain higher photosynthetic activity and nitrogen investment; middle leaves are partially shaded but still contribute substantially to canopy photosynthesis and assimilate accumulation; lower leaves are more strongly affected by shading, senescence, and nitrogen remobilization, and their nitrogen status may reflect canopy senescence, source–sink regulation, and nitrogen redistribution during tuber formation [8]. Therefore, potato LNC often shows a general vertical gradient from the upper to lower canopy layers. Although such a gradient can be described empirically, its magnitude and shape may vary with nitrogen supply, leaf age, shading, canopy structure, and senescence status. Therefore, layer-specific LNC measurements are still necessary to establish ground-truth information on within-canopy nitrogen distribution and to evaluate the statistical association between integrated top-of-canopy hyperspectral reflectance and measured Middle and Bottom LNC. This relationship is important for refined canopy nitrogen diagnosis and canopy functional assessment.

Traditional vegetation indices have been widely used to estimate crop nitrogen-, chlorophyll-, greenness-, and biomass-related traits because of their simple structure, clear physical meaning, and ease of calculation. Common vegetation indices are usually constructed using visible, red-edge, and near-infrared bands, and can characterize canopy greenness, pigment absorption, and structural scattering features [10,11,12]. However, these indices generally rely on a small number of fixed bands, and their sensitivity can be affected by crop type, growth stage, canopy structure, soil background, and environmental conditions [10]. For layer-specific LNC, especially LNC in lower leaves, the target signal is often weakened by upper-leaf occlusion, leaf overlap, shadow effects, and mixed canopy reflectance. As a result, traditional fixed-band indices may not fully capture such attenuated and mixed nitrogen-related spectral information from internal canopy layers. Therefore, it is necessary to expand the sensitive wavelength search space to improve the ability of spectral features to characterize layer-specific LNC.

Two-band spectral indices traverse arbitrary two-band combinations and construct difference, normalized difference, and ratio indices, thereby overcoming the limitation of fixed bands in traditional empirical indices and screening band combinations that are more closely related to target traits across the full spectral range [11]. Compared with traditional vegetation indices, two-band indices improve the flexibility of band selection and enhance data-driven feature extraction to some extent. However, the spectral response of LNC is not determined by only two bands, but is jointly affected by chlorophyll absorption, protein-related biochemical information, leaf water content, dry matter content, internal structure, and canopy architecture [2,3]. For layer-specific LNC in particular, the spectral response is strongly affected by canopy mixing and signal attenuation; thus, two bands alone may be insufficient to fully represent the complex nitrogen response mechanism.

Three-band spectral indices introduce a third wavelength and can integrate information from more spectral regions, thereby enhancing the complementary expression of pigment, red-edge, near-infrared structural, and shortwave infrared biochemical information. Li et al. used optimized three-band spectral indices to estimate canopy nitrogen uptake in corn and wheat, and found that optimized three-band indices generally showed more stable nitrogen prediction ability than existing fixed indices [13]. In recent years, three-band spectral indices have also been used for non-destructive estimation of crop physiological parameters such as chlorophyll, showing good potential for cross-phenology extrapolation and independent testing [14]. These studies suggest that multi-band combinations can provide richer information representation for estimating complex crop physiological parameters. For layer-specific LNC estimation in potato, three-band spectral indices may improve the statistical representation of LNC-related spectral variation by combining visible, red-edge, near-infrared, and shortwave infrared bands. Because top-of-canopy reflectance is an integrated signal affected by multiple canopy layers and structural factors, these indices should be interpreted as empirical tools for linking canopy-scale spectral variation with measured layer-specific LNC. They provide a basis for statistical estimation of Top, Middle, and Bottom LNC, but not for physically separating the reflectance contributions of individual canopy layers.

In addition to spectral index construction, spectral preprocessing is also critical for improving nitrogen estimation accuracy. Fractional-order derivative (FOD) transformation has recently attracted increasing attention in hyperspectral data processing. Compared with original reflectance, FOD can enhance local absorption features, spectral slope variations, and subtle band differences; compared with conventional first- or second-order integer derivatives, FOD provides more flexible regulation between feature enhancement and noise amplification [15,16]. Abulaiti et al. [15] combined fractional-order derivatives with optimized spectral indices to estimate total nitrogen content in cotton canopies and found that FOD treatment improved the ability of spectral features to characterize nitrogen content. Yang et al. also reported that fractional-order derivatives improved the correlation between hyperspectral data and soil total nitrogen content and enhanced model prediction performance [16]. These results indicate that FOD has potential for extracting subtle spectral information and improving the estimation accuracy of nitrogen-related parameters. However, the optimal fractional order may differ among crops, target traits, and canopy layers. At present, the applicability of FOD to layer-specific LNC estimation in potato and its potential gain when combined with three-band spectral indices remain insufficiently understood.

Among hyperspectral modeling methods, partial least squares regression (PLSR) is widely used for estimating leaf nitrogen and other physiological and biochemical parameters because it can handle severe multicollinearity among hyperspectral bands and extract latent variables with maximum covariance with the response variable. Jin et al. reported that PLSR and its variable-selection-based improvements have good application value in retrieving leaf nitrogen content from hyperspectral reflectance, although model performance is still influenced by input variable type, sensitive band selection, and the complexity of the target trait. Therefore, integrating FOD preprocessing, two-band and three-band spectral index construction, and PLSR modeling may provide a more effective technical pathway for high-accuracy estimation of LNC at different potato canopy layers.

Based on a two-year field experiment in the semi-arid region of northern Shaanxi, this study focused on LNC in upper, middle, and lower potato canopy leaves. By integrating hyperspectral reflectance, fractional-order derivative transformation, traditional vegetation indices, two-band spectral indices, three-band spectral indices, and PLSR modeling, this study systematically investigated the capability of hyperspectral data for layer-specific LNC estimation. The specific objectives were to: (1) analyze the vertical distribution characteristics of LNC in upper, middle, and lower potato canopy leaves under different nitrogen fertilizer levels and quantify this vertical pattern using relative LNC ratios and an empirical attenuation coefficient; (2) compare the responses of traditional vegetation indices, two-band spectral indices, and three-band spectral indices to LNC at different canopy layers; (3) evaluate whether integrated top-of-canopy hyperspectral reflectance contains statistical information associated with measured Top, Middle, and Bottom LNC, and compare the performance of traditional vegetation indices, FOD-based two-band indices, and FOD-based three-band indices for layer-specific LNC estimation; and (4) evaluate whether the integration of FOD and three-band spectral indices can improve the prediction accuracy of layer-specific potato LNC, especially for Bottom LNC under conditions of canopy signal attenuation and mixing. This study provides methodological support for describing vertical canopy nitrogen heterogeneity and offers a reference for canopy functional assessment in semi-arid potato systems. Further studies should clarify the biological basis of within-canopy LNC distribution across different growth stages, cultivars, ecological regions, and nitrogen supply conditions.

## 2. Results

### 2.1. Vertical Distribution of Canopy LNC and Its Response to Traditional Vegetation Indices

**Figure 1:**
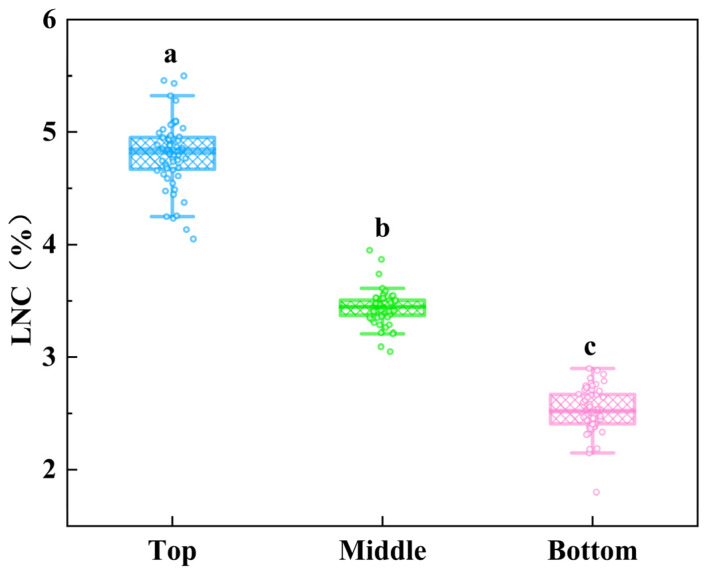
Distribution of leaf nitrogen content in different potato canopy layers. Different lowercase letters indicate significant differences among canopy layers at *p* < 0.05.

One-way ANOVA followed by Tukey’s multiple comparison test showed that LNC differed significantly among the three canopy layers (F = 1587.146, *p* < 0.001). The compact letter display in Figure 1 indicates that Top, Middle, and Bottom LNC were significantly different from each other. The LNC of potato leaves decreased vertically from the Top to Middle and Bottom canopy layers, following the order Top LNC > Middle LNC > Bottom LNC (Figure 1). This vertical gradient indicated that layer-specific LNC measurements provided additional information beyond a single canopy-averaged nitrogen indicator. To further examine whether this vertical LNC pattern was affected by nitrogen fertilizer level, the LNC values of the Top, Middle, and Bottom leaves were summarized separately under each nitrogen treatment (Table 1). Across all nitrogen treatments, Top LNC was consistently higher than Middle and Bottom LNC, whereas Bottom LNC remained the lowest. When Top LNC was used as the reference value, Middle LNC accounted for 70.3–73.8% of Top LNC, and Bottom LNC accounted for 51.9–52.9% of Top LNC. The empirical vertical attenuation coefficient k ranged from 0.318 to 0.328, indicating a relatively stable vertical decline in LNC from the upper to the lower canopy. These results show that strong LNC differences existed among canopy layers, while the relative LNC ratios were comparatively stable across nitrogen fertilizer levels. Since the canopy reflectance was measured from above the canopy, the recorded spectrum represented a mixed top-of-canopy signal, and the individual reflectance contributions of the three leaf layers could not be quantified from these data.

Traditional vegetation indices showed significant relationships with LNC at all three canopy layers, but their response strength varied markedly among layers. In general, the correlations between traditional vegetation indices and LNC were strongest for the top layer, weakened for the middle layer, and were lowest for the bottom layer. This result indicated that traditional fixed-band indices could effectively capture part of the nitrogen variation in upper canopy leaves, but their sensitivity to internal canopy nitrogen status decreased with canopy depth.

For Top LNC, CCI2, SR680, NDVI, CCI1, and SR1 showed relatively strong correlations, with correlation coefficients of 0.770, 0.769, 0.737, 0.737, and 0.728, respectively. OSAVI, SR3, and CI also showed strong responses to Top LNC, suggesting that indices constructed from visible, red-edge, and near-infrared bands could effectively characterize the nitrogen variation in upper canopy leaves.

For Middle LNC, the correlations of traditional vegetation indices decreased compared with those for Top LNC. CCI2 remained the best-performing index, with an r value of 0.632. CCI1, SR680, NDVI, and SR1 followed, with r values of 0.595, 0.579, 0.570, and 0.554, respectively. These results showed that the ability of traditional vegetation indices to characterize LNC weakened when the target leaf layer shifted from the upper canopy to the middle canopy.

For Bottom LNC, the correlations further declined. NDVI, CCI1, and PRI1 showed relatively higher correlations, with r values of 0.524, 0.508, and 0.503, respectively. However, most traditional indices showed lower correlations with Bottom LNC than with Top and Middle LNC. This result indicated that traditional vegetation indices had limited ability to capture the nitrogen variation in lower canopy leaves.

Overall, traditional vegetation indices reflected LNC variation to some extent, but their correlations decreased from the upper to lower canopy layers. The correlation coefficients between traditional vegetation indices and layer-specific LNC are summarized in Table 2.

### 2.2. Correlation Response of Two-Band Spectral Indices Under Different FOD Orders

**Figure 2:**
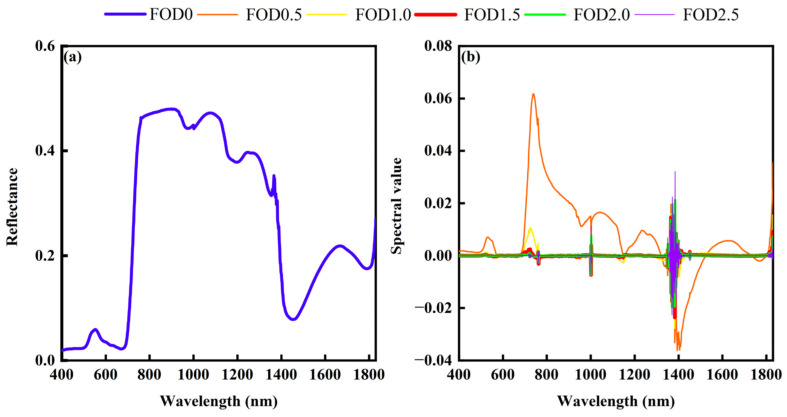
Mean spectral curves under different fractional-order derivative treatments: (**a**) original canopy reflectance spectrum; (**b**) spectra processed using different fractional-order derivative treatments. FOD0 represents the original reflectance spectrum, whereas FOD0.5–FOD2.5 represent spectra processed using fractional-order derivatives.

Compared with traditional vegetation indices, two-band spectral indices expanded the search space of sensitive bands by using all possible two-band combinations. The correlations between DI, NDI, RI, and LNC varied substantially among FOD orders and canopy layers, indicating that fractional-order derivative transformation changed the spectral response of LNC. Figure 2 shows the mean spectral curves after different fractional-order derivative treatments, indicating that FOD transformation altered the spectral amplitude and local variation patterns of the original reflectance spectra.

Under the original reflectance condition, two-band indices already showed stronger correlations with layered LNC than traditional vegetation indices. At FOD0, the selected two-band index for Top LNC was RI, with the selected band combination of 979 and 996 nm and an r value of 0.814. For Middle LNC, the selected two-band index was NDI or RI, with the selected band combination of 977 and 976 nm and an r value of 0.778. For Bottom LNC, the selected two-band index was RI, with the selected band combination of 374 and 350 nm and an r value of −0.618. These results showed that full-band two-band combinations had stronger correlations with LNC than traditional vegetation indices.

After FOD transformation, the correlations between two-band indices and layered LNC increased, with FOD1.5 showing the strongest responses among the tested orders. Under FOD1.5, the highest |r| values reached 0.855, 0.849, and 0.814 for Top, Middle, and Bottom LNC, respectively. The selected band combinations and correlation coefficients of the two-band spectral indices under different FOD orders are listed in Table 3.

**Figure 3:**
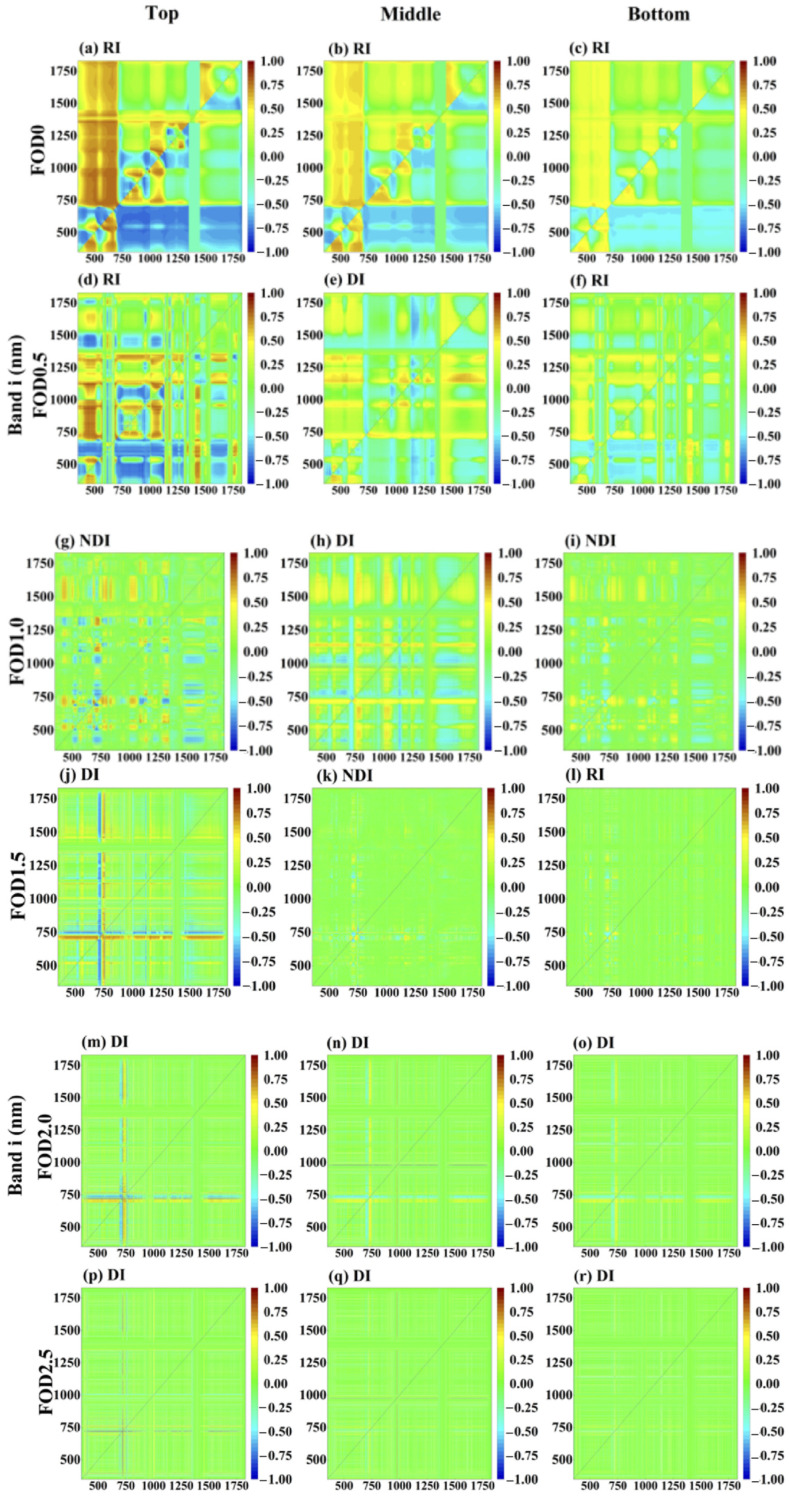
Correlation heatmaps between layer-specific LNC and selected two-band spectral indices under different FOD treatments. The columns represent the Top, Middle, and Bottom canopy layers, respectively, and the rows represent different FOD treatments from FOD0 to FOD2.5. The x- and y-axes indicate band j and band i, respectively. The color scale represents Pearson’s correlation coefficient (r), ranging from −1 to 1. DI, NDI, and RI denote the selected two-band index types for each canopy layer and fractional-order derivative treatment.

The correlation heatmaps further showed that FOD transformation changed the spectral distribution of sensitive band combinations. Under FOD1.5, the high-correlation regions became more concentrated and distinct, especially for Middle and Bottom LNC. Sensitive band combinations were not limited to the conventional visible, red-edge, and near-infrared regions used by traditional vegetation indices, but extended to broader spectral regions, including visible, red-edge, near-infrared, and shortwave infrared wavelengths. Figure 3 presents the correlation patterns under FOD0, FOD0.5, FOD1.0, FOD1.5, FOD2.0, and FOD2.5.

However, increasing the FOD order did not continuously improve the correlations. Under FOD2.0 and FOD2.5, the correlations of some two-band indices decreased. This result indicated that the enhancement effect of FOD was order-dependent, and that excessively high derivative orders may weaken the stability of spectra–LNC relationships. Among the tested FOD orders, FOD1.5 showed the strongest overall correlations for the two-band spectral indices.

### 2.3. Selected Band Combinations of Three-Band Spectral Indices Under Different FOD Orders

Three-band spectral indices showed stronger overall correlations with layered LNC than two-band indices, particularly under FOD1.5.

Under the original reflectance condition, three-band indices already showed relatively high correlations with layered LNC. At FOD0, the highest |r| values reached 0.856, 0.708, and 0.631 for Top, Middle, and Bottom LNC, respectively.

After FOD transformation, FOD1.5 produced the highest correlations across all three canopy layers. Under FOD1.5, the highest |r| values reached 0.893, 0.885, and 0.852 for Top, Middle, and Bottom LNC, respectively.

**Figure 4:**
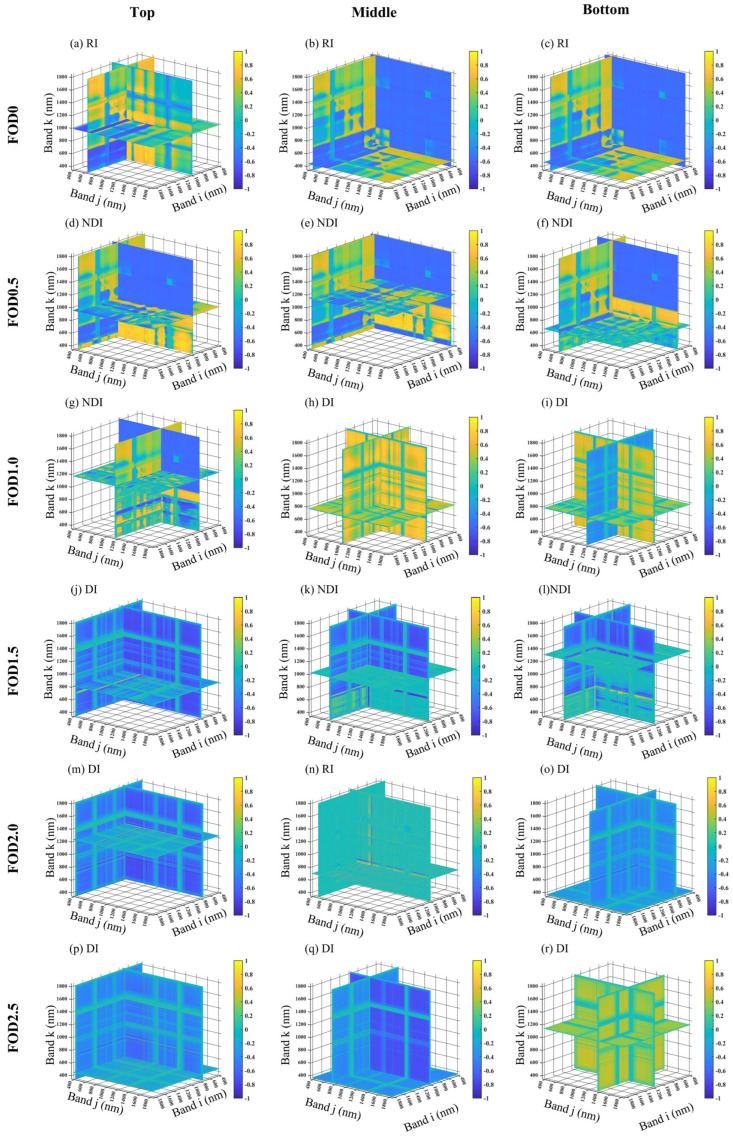
Correlation heatmaps between layer-specific LNC and selected three-band spectral indices under different FOD treatments. The columns represent the Top, Middle, and Bottom canopy layers, respectively, and the rows represent different FOD treatments from FOD0 to FOD2.5. For each heatmap, the displayed slice planes were selected according to the selected three-band combination with the highest absolute Pearson correlation coefficient for the corresponding canopy layer, FOD order, and index type. The axes indicate bands i, j, and k, and the color scale represents Pearson’s correlation coefficient (r). DI, NDI, and RI denote the selected three-band index types for each canopy layer and FOD treatment.

Heat maps of correlation coefficients between the three-band spectral indices and layer-specific LNC are shown in Figure 4. For visualization of the three-band correlation space, the displayed plane in each heatmap was selected by fixing the k-band at the wavelength included in the selected three-band combination with the highest absolute Pearson correlation coefficient for the corresponding canopy layer, FOD order, and index type. The heatmap therefore represents the slice-based correlation distribution over band i and band j under the selected k-band slice. The heatmaps showed layer-dependent correlation patterns, with clearer high-correlation regions under FOD1.5.

The selected band combinations and correlation coefficients of the three-band spectral indices are summarized in Table 4. The selected bands differed among canopy layers; for Bottom LNC, wavelengths around 1142 and 1307 nm were frequently involved.

Overall, the three-band spectral indices showed higher maximum absolute correlations with layered LNC than the corresponding two-band indices, especially under FOD1.5.

### 2.4. Comparison of PLSR Model Performance Among Different Spectral Index Systems

To further evaluate the predictive ability of different spectral index systems, PLSR models were established for Top, Middle, and Bottom LNC using traditional vegetation indices, selected FOD-based two-band indices, and selected FOD-based three-band indices as input variables. For each canopy layer, the 60 samples were divided into a training set and a testing set at a ratio of 2:1, with 40 samples used for model training and 20 samples used for testing.

**Figure 5:**
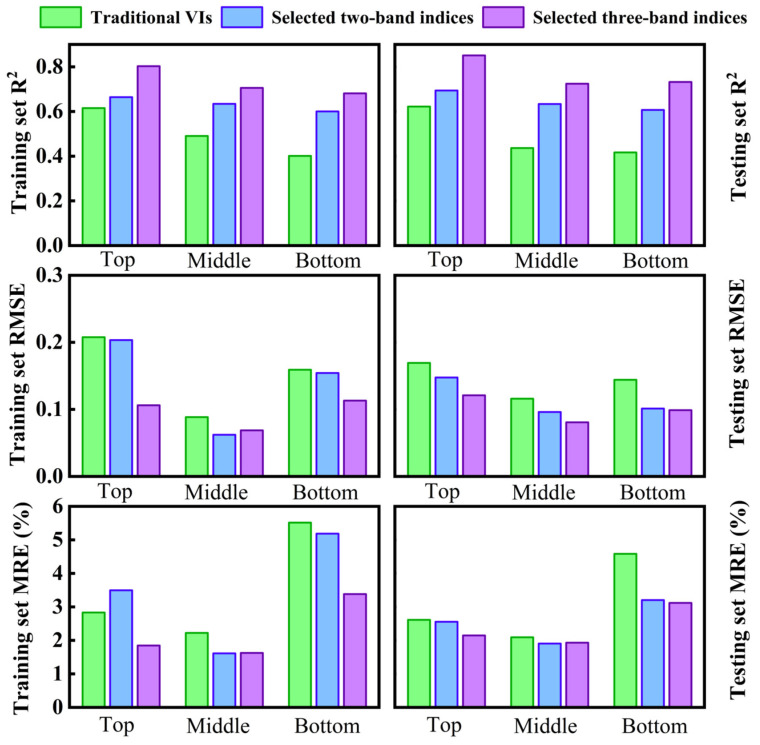
Comparison of model performance for estimating layer-specific LNC using traditional vegetation indices, FOD1.5-based selected two-band spectral indices, and FOD1.5-based selected three-band spectral indices.

As shown in Figure 5, the testing-set R^2^ generally increased from the traditional vegetation index models to the FOD1.5-based two-band and three-band models. Among the three index systems, the FOD1.5-based three-band model achieved the highest testing-set R^2^ for all three canopy layers, although the error-based metrics did not always show the same ranking.

For Top, Middle, and Bottom LNC, the testing-set R^2^ values increased from 0.615, 0.491, and 0.402 in the traditional vegetation index models to 0.803, 0.706, and 0.681 in the FOD1.5-based three-band models, respectively. The largest increase was observed for Bottom LNC, with R^2^ increasing by 0.279 and RMSE decreasing from 0.159 to 0.113. However, RMSE and MRE did not always follow the same ranking as R^2^, indicating that model performance should be evaluated using multiple metrics.

Overall, within the present dataset, the FOD1.5-based three-band indices produced the highest testing-set R^2^ values among the compared index systems, with the largest improvement observed for Bottom LNC. Cross-year validation was further conducted to evaluate the temporal transferability of the selected FOD1.5-based three-band indices. When the 2022 dataset was used for calibration and the 2023 dataset was used for testing, the models achieved R^2^ values of 0.419, 0.326, and 0.237 for Top, Middle, and Bottom LNC, respectively. In the reverse validation, with 2023 used for calibration and 2022 used for testing, the corresponding R^2^ values were 0.367, 0.421, and 0.257. The cross-year validation results were substantially lower than those obtained from the random calibration/testing split. The mean R^2^ decreased from 0.730 under the random calibration/testing split to 0.327 in the 2022-to-2023 validation and 0.348 in the 2023-to-2022 validation. This reduction indicates that the temporal transferability of the selected indices was limited for layer-specific LNC estimation. Therefore, the selected FOD1.5-based three-band indices provide data-supported spectral features for layer-specific LNC estimation under the present experimental conditions, while their stability requires further validation under different years, cultivars, sites, growth stages, and management conditions.

## 3. Discussion

This pattern indicates that nitrogen was not uniformly distributed within the canopy, but was associated with leaf position, light environment, leaf age, and nitrogen remobilization during tuber formation [17,18,19,20,21,22,23,24,25]. Upper leaves generally receive stronger radiation and maintain higher photosynthetic activity, whereas middle and lower leaves are more affected by shading, aging, and source–sink regulation. Therefore, estimating LNC as a single canopy-averaged variable may obscure the nitrogen status of internal canopy layers. The additional nitrogen-level analysis further showed that this vertical gradient was maintained under all nitrogen fertilizer levels, while the relative LNC ratios among layers remained relatively stable. This supports the need to analyze LNC separately for different canopy layers, but also indicates that the empirical attenuation coefficient should be interpreted only as a descriptive index of vertical LNC distribution.

Traditional vegetation indices were able to reflect potato LNC variation to some extent, but their response strength decreased from the upper canopy to the lower canopy. In this study, CCI2, SR680, NDVI, CCI1, and SR1 showed relatively strong correlations with Top LNC, whereas their correlations with Middle LNC and Bottom LNC decreased markedly. This result is consistent with previous findings that vegetation indices mainly characterize canopy greenness, chlorophyll absorption, red-edge variation, and near-infrared structural scattering [25,26,27,28,29,30,31,32,33] SR680 is associated with the red absorption region, NDVI and SR-type indices mainly describe the contrast between red absorption and near-infrared reflectance, and CCI-type indices integrate pigment-sensitive spectral information. Therefore, these indices performed well for upper canopy leaves, where the spectral signal could be directly captured by the sensor. However, for middle and lower canopy leaves, spectral information is affected by upper-leaf occlusion, leaf overlap, shadow effects, and mixed canopy reflectance. As a result, traditional fixed-band indices are insufficient for representing attenuated and mixed nitrogen-related information from internal canopy layers. Similarly, previous studies on rice, maize, and potato have reported that fixed or commonly used vegetation indices for nitrogen estimation are influenced by crop type, canopy structure, background conditions, and growth stage [34,35,36]. Therefore, traditional vegetation indices are more suitable for characterizing upper-canopy nitrogen status, whereas their ability to estimate middle and lower canopy LNC is relatively limited. It should also be noted that most traditional vegetation indices used in this study were originally developed for canopy greenness, chlorophyll content, biomass, or general crop nitrogen status, rather than for layer-specific LNC estimation. Therefore, their lower performance for Middle and Bottom LNC should not be interpreted as a failure of these indices, but rather as evidence that fixed-band empirical indices are not specifically designed to resolve vertically mixed LNC information within a closed potato canopy. In this sense, the traditional indices served as baseline references, whereas the selected two- and three-band indices provided data-supported spectral features for the present layer-specific LNC estimation problem.

All spectral indices used in this study were derived from nadir top-of-canopy reflectance and therefore represented integrated canopy-scale signals rather than layer-specific leaf reflectance. Thus, neither traditional vegetation indices nor the newly constructed FOD-based two-band and three-band indices can directly quantify the optical contributions of Top, Middle, and Bottom leaves. Their use for layer-specific LNC estimation is based on statistical relationships between mixed canopy reflectance and measured layer-specific LNC. The layer-dependent differences in prediction accuracy therefore reflect differences in how strongly Top, Middle, and Bottom LNC were statistically associated with the integrated canopy reflectance signal. The improved performance of FOD-based two-band and three-band indices indicates enhanced representation of LNC-related spectral variation within the mixed canopy signal, rather than physical separation of layer-specific spectral components.

Under the original reflectance condition, two-band indices already showed stronger correlations than traditional indices, indicating that full-band combinations can identify more sensitive wavelength pairs than fixed empirical formulas. This agrees with previous studies showing that narrow-band hyperspectral information and multi-variable spectral features can improve the estimation of crop physiological parameters [30,31,32,33,34,35,36]. However, two-band indices still describe only the spectral relationship between two bands. For a complex physiological trait such as layered LNC, the spectral response is jointly affected by chlorophyll absorption, protein-related biochemical information, leaf water status, dry matter content, internal leaf structure, and canopy architecture [25,26,27,28,29]. These factors are difficult to fully represent using only one or two wavelengths. Therefore, the superior performance of three-band spectral indices in this study suggests that introducing a third wavelength helps integrate information from different spectral regions and enhances the expression of complementary multi-band relationships. In other words, the advantage of three-band spectral indices is not simply due to the increased number of bands, but reflects the fact that layered LNC is controlled by multiple physiological and structural processes that require multidimensional spectral representation.

The selected three-band wavelength combinations further support this interpretation. Under FOD1.5, the selected combination for Top LNC was 764, 399, and 812 nm, involving the visible pigment-sensitive region, the red-edge to near-infrared transition region, and the near-infrared structural scattering region. This combination could simultaneously capture pigment absorption and upper-canopy structural information. For Middle LNC, the selected combination was 979, 710, and 1022 nm. The band around 710 nm is located in the red-edge region and is sensitive to chlorophyll- and nitrogen-related changes, whereas the bands around 979 and 1022 nm may contain information related to water absorption, internal leaf structure, and canopy scattering. For Bottom LNC, the selected combinations included 1142, 724, and 1307 nm, or 1142, 719, and 1307 nm, indicating that the integration of red-edge and shortwave infrared bands played an important role in lower-layer LNC estimation. The red-edge region is closely associated with chlorophyll and nitrogen status, while the shortwave infrared region is more sensitive to leaf water, dry matter, and biochemical composition [27,28,29,30,31]. Since lower leaves are more strongly affected by shading, senescence, and nitrogen redistribution, their LNC variation may be accompanied by changes in leaf structure, water status, and dry matter accumulation. These layer-dependent selected wavelength combinations suggest that the spectral response of LNC is influenced by interactions among leaf nitrogen status, leaf position, biomass distribution, canopy structure, and optical mixing. These wavelength combinations were not randomly selected, but were identified through systematic correlation screening across FOD orders, index types, and canopy layers. Their locations in the visible, red-edge, near-infrared, and shortwave infrared regions are consistent with known spectral responses related to pigment absorption, nitrogen status, leaf water, dry matter, and canopy structure. Therefore, the selected bands provide data-supported and physiologically interpretable spectral features for layer-specific LNC estimation under the present experimental conditions. Nevertheless, their stability and broader applicability still require validation under different cultivars, canopy architectures, layer definitions, growth stages, and environmental conditions. Therefore, Bottom LNC estimation cannot rely solely on greenness or chlorophyll absorption, but requires the integration of spectral signals from red-edge, near-infrared, and shortwave infrared regions. This also explains why three-band spectral indices showed a more pronounced advantage for lower-canopy LNC estimation.

The effectiveness of FOD1.5 can be explained by the balance between spectral feature enhancement and noise control. Original reflectance spectra contain abundant physiological information, but nitrogen-related spectral variations can be attenuated or masked by illumination conditions, background variation, canopy structure, and band redundancy. Fractional-order derivative transformation can enhance local absorption features, spectral slope changes, and subtle differences among adjacent wavelengths while retaining more spectral continuity than integer-order derivatives. In this study, FOD1.5 consistently enhanced the correlations between spectral indices and LNC across all three canopy layers. For two-band indices, FOD1.5-DI, FOD1.5-NDI, and FOD1.5-RI showed strong responses to Top, Middle, and Bottom LNC. For three-band indices, FOD1.5 also produced the highest correlations, with |r| values reaching 0.893, 0.885, and 0.852 for Top, Middle, and Bottom LNC, respectively. Similar studies have shown that fractional-order or optimized spectral transformations can improve hyperspectral estimation of physiological parameters such as nitrogen content, chlorophyll fluorescence, and leaf nitrogen accumulation [37,38,39]. However, when the derivative order increased further to FOD2.0 and FOD2.5, the correlations of some indices decreased, indicating that excessively high derivative orders may amplify spectral noise and random fluctuations, thereby weakening the stability of spectral–LNC relationships. Therefore, FOD1.5 likely provided an effective compromise between preserving biologically meaningful spectral patterns and enhancing subtle local spectral features.

PLSR was an appropriate modeling method for this study because the input variables were derived from hyperspectral bands and their combined indices, which inevitably contain multicollinearity. PLSR can project highly correlated predictors into a small number of latent variables and maximize their covariance with the response variable, making it suitable for hyperspectral modeling under limited sample sizes [40,41]. The objective of this study was not to pursue the highest possible prediction accuracy using complex machine learning algorithms, but to compare the information representation ability of traditional indices, two-band FOD indices, and three-band FOD indices under a consistent modeling framework. Therefore, using PLSR reduced the influence of model complexity on result interpretation and allowed the performance differences among index systems to be more reasonably attributed to the enhancement of spectral information itself. The results showed that the testing-set R^2^ increased from traditional indices to FOD-based two-band indices and then to FOD-based three-band indices, whereas RMSE and MRE showed some layer-dependent variation. Therefore, the advantage of FOD1.5-based two-band indices should be interpreted mainly as improved explanatory ability and enhanced spectral information representation, rather than a uniform improvement across all error metrics.

Among all models, the FOD1.5-based three-band model achieved the highest testing R^2^ for all three canopy layers, with values of 0.803, 0.706, and 0.681 for Top, Middle, and Bottom LNC, respectively. This indicates that the selected three-band FOD indices contained stable and effective information for layered LNC estimation.

The largest improvement was observed for Bottom LNC, but this should not be interpreted as direct optical measurement of lower canopy leaves. Because reflectance was measured from above the canopy in the nadir direction, the recorded signal was an integrated canopy-scale reflectance affected by upper leaves, lower leaves, leaf angles, shadows, canopy gaps, and background effects. The improved Bottom LNC prediction therefore indicates enhanced statistical sensitivity to canopy-scale spectral variations associated with lower-layer nitrogen status [19,20,21], rather than quantitative separation of the optical contribution of lower leaves. Resolving the individual contributions of different canopy layers would require additional measurements, such as layer-specific reflectance, within-canopy light interception, leaf area distribution, or radiative transfer modeling.

Several limitations should be acknowledged. First, the dataset was obtained from two years, one site, one cultivar, and one main growth period; therefore, the stability of the selected wavelength combinations and FOD order requires further validation before broader application. The cross-year validation results further showed limited temporal generalization, indicating that year-specific canopy structure, illumination, soil background, and nitrogen distribution may influence model transferability [42]. Second, the ground-truth measurements were limited to layer-specific LNC, whereas additional variables such as chlorophyll content, leaf water content, leaf area distribution, biomass allocation, and within-canopy light interception would help clarify the physiological and structural links between LNC and canopy reflectance. Third, this study used PLSR to compare spectral index systems, but future work may evaluate nonlinear models and radiative transfer approaches to improve both prediction accuracy and mechanistic interpretation.

Overall, this study indicates that layer-specific LNC estimation provides a more refined description of potato canopy nitrogen status than canopy-averaged approaches. Traditional vegetation indices mainly reflected upper-canopy nitrogen variation, whereas FOD1.5 combined with three-band spectral indices improved the statistical estimation of Top, Middle, and Bottom LNC from integrated canopy reflectance [38,39,40,41,42,43]. These findings provide a methodological reference for assessing vertical canopy nitrogen status and functional heterogeneity in potato. Further studies should clarify the biological basis of within-canopy LNC distribution across different years, cultivars, growth stages, ecological regions, and nitrogen supply conditions [44,45,46].

## 4. Materials and Methods

### 4.1. Study Area and Experimental Design

The field experiment was conducted from 2022 to 2023 at the Potato Experimental Demonstration Station of Northwest A&F University in Yulin, Shaanxi Province, China. The experimental site is located in Yulin City, Shaanxi Province, at 38°23′ N and 109°43′ E. This region belongs to a typical arid and semi-arid climate zone, with a long-term mean annual precipitation of approximately 371 mm. The soil type at the experimental site is dominated by sandy soil, representing a typical potato production region in northwestern China.

The potato cultivar used in this study was ‘Qingshu 9’. The experiment included five nitrogen application levels and two biochar application levels. The nitrogen treatments were N0, N1, N2, N3, and N4, corresponding to 0, 90, 180, 270, and 360 kg N ha^−1^, respectively. The biochar treatments were B0 and B1, corresponding to 0 and 30 t ha^−1^, respectively. The biochar was provided by Tianjin Bormai Environmental Technology Co., Ltd. (Tianjin, China). A total of 10 treatments were established, with three replicates for each treatment, resulting in 30 experimental plots. The same treatment layout and three plot replicates were maintained in both experimental years, and the plot was used as the experimental unit for subsequent LNC measurement and canopy spectral analysis. Each plot was 4 m × 12 m, with an area of 48 m^2^. The plots were arranged randomly, and 3 m buffer rows were established around the experimental area.

Potatoes were planted on 5 May 2022 and 1 May 2023, respectively. Biochar was applied once before planting in 2022 according to the treatment design and was not reapplied in 2023, in order to preserve the residual field effect of the initial biochar application. The application rates of phosphorus and potassium fertilizers were kept consistent across all plots, at 60 kg ha^−1^ and 225 kg ha^−1^, respectively. Nitrogen, phosphorus, and potassium fertilizers were supplied as urea, calcium superphosphate (16% P_2_O_5_), and potassium chloride (0-52-0), respectively. Fertilizers were applied in furrows before planting, approximately 25 cm away from the plants. Other field management practices were consistent with local potato production practices.

### 4.2. Stratified Canopy Leaf Sampling and LNC Measurement

To characterize the vertical heterogeneity of nitrogen within the potato canopy, stratified leaf sampling was conducted during the tuber formation to early tuber bulking period. Sampling was performed on 7 July 2022 and 8 July 2023, corresponding to 63 and 68 days after planting, respectively, and was synchronized with canopy spectral data acquisition. In each plot, three representative plants with uniform growth and without visible disease, pest damage, or mechanical injury were randomly selected. Leaves were divided into three canopy layers along the vertical direction: upper, middle, and lower layers, hereafter referred to as Top, Middle, and Bottom, respectively. Because potato plants often have multiple stems and uneven shoot development, the layer classification was based on the relative vertical position of leaves within the whole plant canopy rather than on the leaf order of a single stem. Top leaves were defined as fully expanded and well-illuminated functional leaves located in the upper canopy. Middle leaves were fully expanded leaves located in the central canopy and partially affected by shading. Bottom leaves were older but still physiologically functional leaves located in the lower canopy, where shading and early senescence effects were more pronounced. For each plot, leaves from the same canopy layer across the selected representative plants were pooled to form one layer-specific sample.

**Figure 6:**
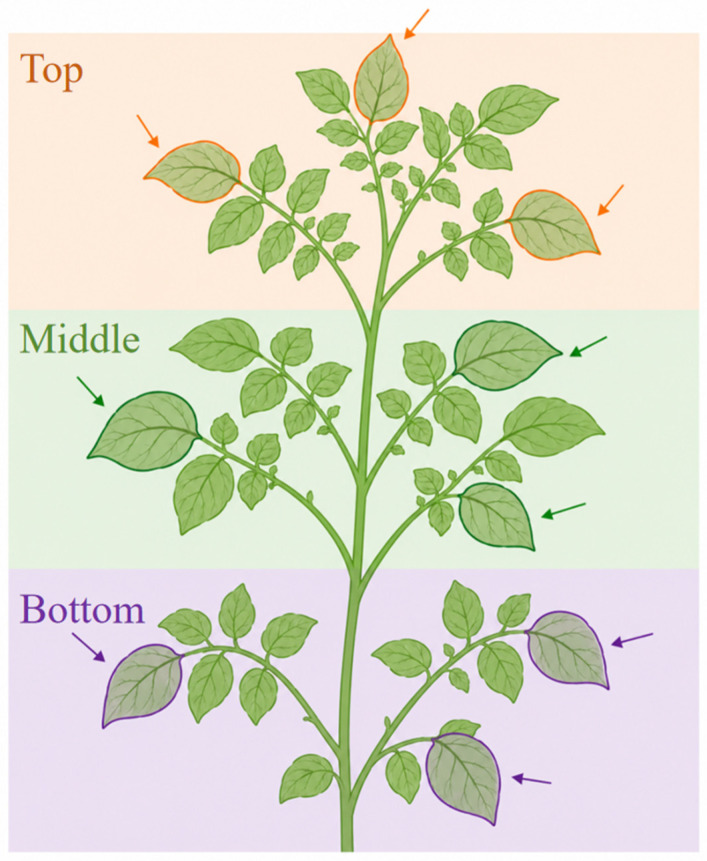
Schematic diagram of stratified leaf sampling in the potato canopy. Arrows indicate the representative leaves sampled from the Top, Middle, and Bottom canopy layers.

The stratified leaf sampling scheme is shown in Figure 6, in which the potato canopy was divided into Top, Middle, and Bottom layers along the vertical direction for layer-specific LNC determination.

Thus, 30 samples were obtained for each canopy layer in each year, and 60 samples were obtained for each layer across the two years. In total, 180 LNC samples were collected from the three canopy layers.

After collection, leaf samples were transported to the laboratory, washed with tap water to remove surface dust, rinsed with deionized water, and then dried with absorbent paper. The samples were then enzyme-inactivated, oven-dried to constant weight, ground, and sieved for subsequent analysis. Leaf total nitrogen content was determined using a conventional plant nitrogen determination method and calculated on a dry matter basis as leaf nitrogen content, namely LNC [47]. Finally, Top LNC, Middle LNC, and Bottom LNC were obtained and used to analyze the vertical distribution characteristics of nitrogen within the potato canopy and its spectral response patterns.

### 4.3. Canopy Hyperspectral Data Acquisition and Preprocessing

Canopy hyperspectral data were acquired simultaneously with leaf sampling on 7 July 2022 and 8 July 2023. Measurements were conducted under clear, cloudless, low-wind, and stable illumination conditions. Spectral measurements were taken as close as possible to solar noon to reduce the influence of changes in solar zenith angle on canopy reflectance.

A portable ASD FieldSpec 3 field spectroradiometer (Analytical Spectral Devices, Inc., Boulder, CO, USA) was used to collect potato canopy reflectance data, with the spectral range of 350–1830 nm used for subsequent analysis. Before each measurement, the instrument was calibrated using a standard white reference panel, and white-panel calibration was repeated during data acquisition when illumination conditions changed [48,49]. The spectral probe was held approximately 50–80 cm above the top of the potato canopy and positioned vertically downward toward the plot canopy, avoiding plot edges and areas with obvious missing plants, to ensure that the observed area represented the overall canopy condition of each plot. In each plot, three representative measurement positions were selected, and three spectral curves were collected at each position. Abnormal curves were first removed. The remaining curves were smoothed using the Savitzky–Golay method with a second-order polynomial and an 11-point moving window to reduce high-frequency spectral noise [50]. The smoothed curves were then averaged first at the position level and then at the plot level to obtain plot-scale canopy reflectance. These within-plot spectral measurements were treated as technical replicates and were not used as independent biological samples. Because the hyperspectral measurements were acquired using a non-imaging spectrometer positioned above the canopy in the nadir direction, the measured reflectance represented a plot-scale top-of-canopy mixed signal. This signal integrated reflectance components from different leaf layers, leaf angles, shadowed leaves, canopy gaps, and background effects, rather than layer-specific reflectance from Top, Middle, or Bottom leaves. Therefore, the spectral models developed in this study were used to evaluate statistical relationships between mixed canopy reflectance and measured layer-specific LNC, but they were not intended to quantitatively separate the individual reflectance contributions of different canopy layers. Since this study focused on the responses of traditional vegetation indices, two-band spectral indices, and three-band spectral indices to layer-specific LNC, all subsequent index calculations were based on the preprocessed canopy hyperspectral reflectance.

### 4.4. Fractional-Order Derivative Spectral Transformation

Fractional-order derivative transformation was applied to the preprocessed canopy reflectance.

In this study, multiple fractional orders were used to transform canopy reflectance. The FOD orders were set to 0, 0.5, 1.0, 1.5, 2.0, and 2.5. The 0-order spectrum represents the original reflectance spectrum, while the other orders represent fractional-order derivative treatments with different transformation intensities. The fractional-order derivative was calculated discretely using the Grünwald–Letnikov definition, expressed as follows [51]:

(1) $$D^{\alpha }f(\lambda_{i})=\frac{1}{h^{\alpha }}\sum_{k=0}^{m}(-1{)}^{k}\frac{\Gamma (\alpha +1)}{k!\Gamma (\alpha -k+1)}f(\lambda_{i}-kh)$$

where D^α^f(λ_i_) is the fractional-order derivative value at λ_i_; α is the fractional order; h is the spectral sampling interval; *k* is the summation index, ranging from 0 to m; m is the number of preceding bands involved in the calculation; Γ(·) is the Gamma function; and f(λ_i_ − *kh*) represents the spectral reflectance at the corresponding wavelength.

Through FOD treatments at different orders, multiple fractional-order derivative spectra were obtained and used for subsequent construction of two-band and three-band spectral indices. The correlation and modeling results under different orders were used to determine the most suitable spectral enhancement order.

### 4.5. Construction of Traditional Vegetation Indices

To compare the performance of empirical fixed-band indices and full-band combination indices in estimating layer-specific LNC, 12 commonly used vegetation indices were first selected as the traditional index system. Traditional vegetation indices are mainly constructed using visible, red-edge, and near-infrared bands, and can reflect canopy greenness, chlorophyll absorption, and vegetation structural variation [52,53,54,55,56,57]. Based on previous studies and the requirements of potato canopy nitrogen diagnosis, the selected indices included the normalized difference vegetation index (NDVI), normalized difference red-edge index (NDRE), optimized soil-adjusted vegetation index (OSAVI), green normalized difference vegetation index (GNDVI), chlorophyll index (CI), chlorophyll content indices (CCI1 and CCI2), photochemical reflectance index (PRI1), simple ratio indices (SR1 and SR3), and simple ratio indices centered at 705 and 680 nm (SR705 and SR680), which are closely related to chlorophyll or nitrogen status [52,53,54,55,56,57].

All traditional vegetation indices were calculated using the original canopy reflectance. After calculation, the correlations between the 12 traditional vegetation indices and Top LNC, Middle LNC, and Bottom LNC were analyzed separately to evaluate the response ability of fixed-band empirical indices to LNC at different canopy layers.

### 4.6. Construction of Two-Band Spectral Indices

To overcome the dependence of traditional vegetation indices on fixed bands, two-band spectral indices were constructed by traversing all possible two-band combinations across the full spectral range. These indices expanded the sensitive wavelength search space by applying difference, normalized difference, and ratio operations to arbitrary band pairs, thereby allowing band combinations more closely related to layer-specific LNC to be identified [11,12].

For each FOD order, the difference index (DI), normalized difference index (NDI), and ratio index (RI) were calculated as follows:

(2) $$DI_{i,j}=R_{i}-R_{j}$$

(3) $$NDI_{i,j}=\frac{R_{i}-R_{j}}{R_{i}+R_{j}}$$

(4) $$RI_{i,j}=\frac{R_{i}}{R_{j}}$$

where R_i_ and R_j_ represent the reflectance or fractional-order derivative spectral values at the i-th and j-th bands, respectively; DI_i,j_, NDI_i,j_ and RI_i,j_ represent the difference index, normalized difference index, and ratio index constructed from two bands, respectively.

For each canopy layer, FOD order, and index type, Pearson correlation coefficients were calculated between the two-band spectral indices and Top LNC, Middle LNC, and Bottom LNC. The band combination with the highest absolute correlation coefficient was retained as the selected two-band spectral index under the corresponding condition, and its band positions, index type, and correlation coefficient were recorded.

### 4.7. Construction of Three-Band Spectral Indices

On the basis of the two-band spectral indices, a third band was introduced to construct three-band spectral indices. The purpose was to enhance the multi-band representation of spectral information related to layer-specific LNC. Compared with two-band combinations, three-band spectral indices can integrate information from three wavelengths and may better characterize the combined effects of pigment absorption, red-edge variation, near-infrared structural scattering, and shortwave-infrared biochemical features [13,14,58,59].

In this study, three-band difference indices, three-band normalized difference indices, and three-band ratio indices were constructed. Their general forms were expressed as follows:

(5) $$3D-DI=R_{i}-R_{j}-R_{k}$$

(6) $$3D-NDI=\frac{R_{i}-R_{j}}{R_{i}+R_{j}+R_{k}}$$

(7) $$3D-RI=\frac{R_{i}}{R_{j}+R_{k}}$$

where R_i_, R_j_, and R_k_ represent the reflectance or fractional-order derivative spectral values at the i-th, j-th, and k-th bands, respectively; DI, NDI, and RI represent the three-band difference index, three-band normalized difference index, and three-band ratio index, respectively.

In this study, sensitive bands refer to the wavelengths whose reflectance or fractional-order derivative values showed relatively strong Pearson correlations with layer-specific LNC under a given FOD order and canopy layer. For each FOD order, candidate three-band combinations were screened from the top K sensitive bands (K = 200). The correlation coefficients between the three types of three-band spectral indices and Top LNC, Middle LNC, and Bottom LNC were then calculated separately. For each canopy layer, the three-band combination with the highest absolute correlation coefficient was retained as the selected three-band spectral index, and its band positions, index type, and correlation coefficient were recorded.

### 4.8. Correlation Analysis and Sensitive Index Selection

To identify spectral variables sensitive to layer-specific LNC, Pearson correlation coefficients were calculated between traditional vegetation indices, two-band spectral indices, three-band spectral indices, and Top LNC, Middle LNC, and Bottom LNC [60]. Correlation analysis was conducted separately for different canopy layers and different FOD orders.

For two-band spectral indices, all possible two-band combinations were traversed under each FOD order, and the selected band combinations were determined according to the maximum absolute correlation coefficient. For three-band spectral indices, candidate three-band combinations were screened from the top K sensitive bands under each FOD order, and the selected combinations were then determined based on the maximum absolute correlation coefficient. A larger absolute correlation coefficient indicated a stronger response of the index to the corresponding layer-specific LNC.

By comparing correlations among different FOD orders, index types, and canopy layers, the ability of FOD treatment to enhance the spectral response of layer-specific LNC was assessed, and the effects of two-band and three-band spectral indices on the characterization of middle and lower canopy LNC were further evaluated.

### 4.9. Model Construction and Evaluation

To further evaluate the predictive ability of different spectral index systems for layer-specific potato LNC, partial least squares regression (PLSR) models were constructed for Top LNC, Middle LNC, and Bottom LNC. PLSR can extract latent variables under conditions of high-dimensional predictors and strong collinearity among variables, and can establish stable relationships between spectral features and target traits [61]. Therefore, it is suitable for estimating crop physiological parameters based on hyperspectral band combinations and spectral indices.

For each canopy layer, three types of PLSR models were established. The first type used the 12 traditional vegetation indices as input variables to represent the baseline estimation ability of fixed empirical indices. The second type used the selected two-band spectral indices identified from the correlation-response analysis as input variables to evaluate the estimation ability of full-band two-band combinations after FOD treatment. The third type used the selected three-band spectral indices identified from the correlation-response analysis as input variables to examine the contribution of three-band synergistic spectral information to layer-specific LNC estimation. For the two-band and three-band index models, three index variables were included for each canopy layer, namely DI, NDI, and RI. Specifically, for each canopy layer and FOD order, the two-band DI, NDI, and RI with the highest absolute Pearson correlation coefficients with LNC were retained as the selected two-band indices. Similarly, the three-band DI, NDI, and RI with the highest absolute Pearson correlation coefficients with LNC were retained as the selected three-band indices. Therefore, each two-band PLSR model and each three-band PLSR model used three selected spectral indices as input variables.

For model construction and evaluation, the 60 samples from each canopy layer were divided into a calibration set and a testing set at a ratio of 2:1, with 40 samples used for model calibration and 20 samples used for testing. The testing set was used to evaluate the predictive performance of the calibrated models under the same input-variable selection and sample partitioning strategy. To ensure comparability among different index systems, all models used the same sample partitioning scheme and evaluation metrics. In addition to the random calibration/testing split, cross-year validation was conducted to evaluate the temporal transferability of the selected FOD1.5-based three-band indices. The 2022 dataset was used for sensitive-band selection, three-band DI, three-band NDI, and three-band RI construction, and PLSR calibration, and the independent 2023 dataset was used for testing. The reverse validation, with the 2023 dataset used for index selection and model calibration and the 2022 dataset used for testing, was also performed as a sensitivity analysis. For each PLSR model, the number of latent variables was selected using leave-one-out cross-validation within the calibration dataset. Differences in LNC among the Top, Middle, and Bottom canopy layers were tested using one-way analysis of variance (ANOVA), followed by Tukey’s multiple comparison test, using IBM SPSS Statistics version 27.0 (IBM Corp., Armonk, NY, USA). Significant differences were indicated using compact letter displays at *p* < 0.05. To further describe the nitrogen-level-dependent vertical distribution of LNC, Top, Middle, and Bottom LNC were summarized separately for each nitrogen fertilizer level. For each nitrogen level, the relative LNC ratios of the middle and bottom layers to the top layer were calculated as Middle/Top (%) and Bottom/Top (%), respectively. These ratios were used to evaluate the proportional relationship of LNC among canopy layers under different nitrogen supplies. In addition, an empirical vertical attenuation coefficient was calculated using the log-linear model ln(LNC_layer) = a − k × Layer, where Layer was coded as 0, 1, and 2 for Top, Middle, and Bottom leaves, respectively. The coefficient k was used as a descriptive indicator of the steepness of the vertical LNC decline from the upper to the lower canopy.

Model accuracy was evaluated using the coefficient of determination (R^2^), root mean square error (RMSE), and mean relative error (MRE) [62], calculated as follows:

(8) $$R^{2}=\frac{\sum_{i=1}^{n}{\left(\hat{y_{i}}-\bar{y}\right)}^{2}}{\sum_{i=1}^{n}{\left(y_{i}-\bar{y}\right)}^{2}}$$

(9) $$RMSE=\sqrt{\frac{\sum_{i=1}^{n}{\left(y_{i}-\bar{y}\right)}^{2}}{n}}$$

(10) $$MRE=\frac{1}{n}\sum_{i=1}^{n}\frac{\left|{\hat{y}}_{i}-y_{i}\right|}{y_{i}}\times 100\%$$

In the formula:

$\hat{y_{i}}$ represents the predicted value of the model;

$y_{i}$ represents the actual sampled value;

$\bar{y}$ represents the average value;

n represents the number of samples.

## 5. Conclusions

This study developed a layer-specific LNC estimation framework for potato canopy by integrating canopy hyperspectral reflectance, FOD transformation, two-band and three-band spectral indices, and PLSR modeling. The results showed that potato canopy LNC exhibited a clear vertical gradient, following the order Top LNC > Middle LNC > Bottom LNC. These results suggest that combining spectral assessment of Top LNC with the observed relative LNC ratios among canopy layers, namely Top = 100, Middle ≈ 72.5, and Bottom ≈ 52.5, may be a promising approach for describing the vertical LNC distribution in potato canopies. Compared with traditional vegetation indices, FOD-based two-band indices improved the spectral response to layer-specific LNC, and FOD1.5 showed the strongest overall performance among the tested derivative orders.

Three-band spectral indices further improved the representation of layer-specific LNC. The FOD1.5-based three-band model achieved the highest testing-set R^2^ values among the compared index systems, with values of 0.803, 0.706, and 0.681 for Top, Middle, and Bottom LNC, respectively. The largest improvement was observed for Bottom LNC, suggesting that FOD1.5 combined with three-band spectral indices can improve the statistical estimation of layer-specific LNC from integrated canopy reflectance.

However, the improved Bottom LNC estimation should not be interpreted as direct optical measurement of lower canopy leaves or quantitative separation of their reflectance contribution. In addition, cross-year validation showed reduced prediction accuracy compared with the random calibration/testing split, indicating limited temporal generalization of the selected indices. Therefore, the proposed indices provide data-supported spectral features for layer-specific LNC estimation, and the framework provides a methodological reference for refined canopy nitrogen diagnosis in potato.

## Figures and Tables

**Table 1:** Leaf nitrogen content and relative LNC ratios among canopy layers under different nitrogen fertilizer levels.

Nitrogen Level	Top LNC (%)	Middle LNC (%)	Bottom LNC (%)	Middle/Top (%)	Bottom/Top (%)	k
N0	4.638 ± 0.385	3.421 ± 0.223	2.407 ± 0.227	73.8	51.9	0.328
N1	4.760 ± 0.318	3.381 ± 0.136	2.494 ± 0.238	71.0	52.4	0.323
N2	4.940 ± 0.232	3.472 ± 0.106	2.599 ± 0.172	70.3	52.6	0.321
N3	4.847 ± 0.256	3.464 ± 0.151	2.564 ± 0.151	71.5	52.9	0.318
N4	4.890 ± 0.230	3.478 ± 0.077	2.572 ± 0.141	71.1	52.6	0.321

Note: Values are means ± SD, *n* = 12. N0, N1, N2, N3, and N4 represent 0, 90, 180, 270, and 360 kg N ha^−1^, respectively. Middle/Top and Bottom/Top were calculated based on the mean LNC values of each canopy layer. The vertical attenuation coefficient k was calculated using the empirical log-linear model ln(LNC_layer) = a − k × Layer, where Layer = 0, 1, and 2 for Top, Middle, and Bottom leaves, respectively.

**Table 2:** Correlation coefficients between traditional vegetation indices and layer-specific leaf nitrogen content.

Traditional Index	Top LNC	Middle LNC	Bottom LNC
PRI1	0.546	0.438	0.503
NDVI	0.737	0.570	0.524
CI	0.662	0.463	0.367
CCI1	0.737	0.595	0.508
SR1	0.728	0.554	0.399
SR3	0.688	0.514	0.417
SR680	0.769	0.579	0.460
SR705	0.645	0.466	0.330
CCI2	0.770	0.632	0.482
NDRE	−0.317	−0.393	−0.315
GNDVI	0.600	0.381	0.365
OSAVI	0.715	0.437	0.452

Note: All correlation coefficients are statistically significant at *p* < 0.05. Top LNC, Middle LNC, and Bottom LNC represent the upper, middle, and lower canopy layers, respectively.

**Table 3:** Selected band combinations and correlation coefficients of two-band spectral indices for layer-specific LNC under different fractional-order derivative treatments.

Order	Layer	Index Type	Selected Bands (i, j)/nm	r
FOD0	Top	DI	(1662, 1349)	−0.804
FOD0	Top	NDI	(996, 979)	−0.801
FOD0	Top	RI	(979, 996)	0.814
FOD0	Middle	DI	(977, 976)	0.752
FOD0	Middle	NDI	(977, 976)	0.778
FOD0	Middle	RI	(977, 976)	0.778
FOD0	Bottom	DI	(374, 350)	−0.598
FOD0	Bottom	NDI	(374, 350)	−0.612
FOD0	Bottom	RI	(374, 350)	−0.618
FOD0.5	Top	DI	(1606, 950)	−0.823
FOD0.5	Top	NDI	(932, 478)	0.833
FOD0.5	Top	RI	(934, 446)	0.833
FOD0.5	Middle	DI	(979, 978)	−0.822
FOD0.5	Middle	NDI	(977, 976)	0.783
FOD0.5	Middle	RI	(978, 979)	0.798
FOD0.5	Bottom	DI	(1142, 1141)	0.630
FOD0.5	Bottom	NDI	(1778, 1336)	0.635
FOD0.5	Bottom	RI	(1336, 1778)	−0.644
FOD1.0	Top	DI	(804, 676)	−0.835
FOD1.0	Top	NDI	(1463, 804)	0.847
FOD1.0	Top	RI	(804, 1182)	0.847
FOD1.0	Middle	DI	(977, 900)	0.825
FOD1.0	Middle	NDI	(977, 735)	0.771
FOD1.0	Middle	RI	(977, 497)	0.820
FOD1.0	Bottom	DI	(1143, 550)	0.661
FOD1.0	Bottom	NDI	(1345, 1093)	0.663
FOD1.0	Bottom	RI	(1143, 1338)	−0.662
FOD1.5	Top	DI	(1118, 764)	0.855
FOD1.5	Top	NDI	(1116, 764)	−0.800
FOD1.5	Top	RI	(1116, 764)	−0.811
FOD1.5	Middle	DI	(979, 399)	−0.828
FOD1.5	Middle	NDI	(979, 717)	−0.849
FOD1.5	Middle	RI	(979, 801)	0.837
FOD1.5	Bottom	DI	(1165, 1142)	−0.761
FOD1.5	Bottom	NDI	(1142, 764)	−0.808
FOD1.5	Bottom	RI	(1142, 718)	0.814
FOD2.0	Top	DI	(732, 399)	−0.846
FOD2.0	Top	NDI	(732, 350)	−0.764
FOD2.0	Top	RI	(732, 350)	−0.764
FOD2.0	Middle	DI	(977, 909)	0.812
FOD2.0	Middle	NDI	(977, 351)	−0.776
FOD2.0	Middle	RI	(977, 693)	0.786
FOD2.0	Bottom	DI	(1142, 733)	0.728
FOD2.0	Bottom	NDI	(1010, 699)	−0.651
FOD2.0	Bottom	RI	(1142, 670)	0.673
FOD2.5	Top	DI	(725, 398)	−0.838
FOD2.5	Top	NDI	(725, 350)	−0.781
FOD2.5	Top	RI	(725, 350)	−0.782
FOD2.5	Middle	DI	(978, 723)	−0.803
FOD2.5	Middle	NDI	(978, 352)	−0.754
FOD2.5	Middle	RI	(978, 721)	0.780
FOD2.5	Bottom	DI	(1344, 1136)	−0.721
FOD2.5	Bottom	NDI	(1625, 944)	−0.639
FOD2.5	Bottom	RI	(1145, 742)	−0.622

Note: Values in parentheses represent the selected band combination (i, j). All correlation coefficients are statistically significant at *p* < 0.05.

**Table 4:** Selected band combinations and correlation coefficients of three-band spectral indices under different fractional-order derivative treatments.

Order	Layer	Index Type	Selected Bands (i, j, k)/nm	r
FOD0	Top	Three-band DI	(729, 633, 726)	0.776
FOD0	Top	Three-band NDI	(657, 935, 779)	−0.828
FOD0	Top	Three-band RI	(961, 625, 988)	0.856
FOD0	Middle	Three-band DI	(660, 666, 658)	0.598
FOD0	Middle	Three-band NDI	(443, 436, 695)	−0.700
FOD0	Middle	Three-band RI	(443, 442, 436)	−0.708
FOD0	Bottom	Three-band DI	(725, 693, 722)	0.496
FOD0	Bottom	Three-band NDI	(471, 460, 746)	−0.589
FOD0	Bottom	Three-band RI	(471, 479, 461)	−0.631
FOD0.5	Top	Three-band DI	(933, 567, 446)	0.841
FOD0.5	Top	Three-band NDI	(946, 454, 952)	0.873
FOD0.5	Top	Three-band RI	(934, 633, 446)	0.868
FOD0.5	Middle	Three-band DI	(445, 417, 572)	0.660
FOD0.5	Middle	Three-band NDI	(440, 419, 1145)	−0.781
FOD0.5	Middle	Three-band RI	(443, 464, 415)	−0.736
FOD0.5	Bottom	Three-band DI	(484, 664, 565)	0.614
FOD0.5	Bottom	Three-band NDI	(637, 635, 662)	0.665
FOD0.5	Bottom	Three-band RI	(666, 565, 564)	−0.587
FOD1.0	Top	Three-band DI	(446, 894, 1517)	−0.869
FOD1.0	Top	Three-band NDI	(803, 1125, 1168)	0.876
FOD1.0	Top	Three-band RI	(804, 1180, 1174)	0.861
FOD1.0	Middle	Three-band DI	(977, 979, 768)	0.866
FOD1.0	Middle	Three-band NDI	(977, 768, 1147)	−0.847
FOD1.0	Middle	Three-band RI	(977, 1569, 899)	0.798
FOD1.0	Bottom	Three-band DI	(1143, 1142, 776)	0.738
FOD1.0	Bottom	Three-band NDI	(1143, 793, 1142)	−0.702
FOD1.0	Bottom	Three-band RI	(1143, 771, 1142)	−0.710
FOD1.5	Top	Three-band DI	(764, 399, 812)	−0.893
FOD1.5	Top	Three-band NDI	(764, 1118, 382)	0.854
FOD1.5	Top	Three-band RI	(705, 1154, 695)	0.836
FOD1.5	Middle	Three-band DI	(979, 399, 1614)	−0.880
FOD1.5	Middle	Three-band NDI	(979, 710, 1022)	−0.885
FOD1.5	Middle	Three-band RI	(979, 1598, 524)	−0.881
FOD1.5	Bottom	Three-band DI	(1142, 789, 1165)	0.820
FOD1.5	Bottom	Three-band NDI	(1142, 724, 1307)	0.852
FOD1.5	Bottom	Three-band RI	(1142, 719, 1307)	0.852
FOD2.0	Top	Three-band DI	(720, 399, 1234)	−0.870
FOD2.0	Top	Three-band NDI	(732, 1530, 827)	0.804
FOD2.0	Top	Three-band RI	(758, 733, 1094)	−0.818
FOD2.0	Middle	Three-band DI	(977, 720, 386)	0.854
FOD2.0	Middle	Three-band NDI	(979, 1070, 717)	−0.818
FOD2.0	Middle	Three-band RI	(977, 475, 697)	0.859
FOD2.0	Bottom	Three-band DI	(732, 1142, 366)	−0.805
FOD2.0	Bottom	Three-band NDI	(732, 1142, 731)	0.754
FOD2.0	Bottom	Three-band RI	(1142, 703, 862)	0.759
FOD2.5	Top	Three-band DI	(725, 398, 447)	−0.883
FOD2.5	Top	Three-band NDI	(723, 1518, 384)	0.792
FOD2.5	Top	Three-band RI	(725, 721, 752)	0.777
FOD2.5	Middle	Three-band DI	(978, 723, 365)	−0.875
FOD2.5	Middle	Three-band NDI	(978, 723, 980)	−0.774
FOD2.5	Middle	Three-band RI	(977, 716, 1213)	0.832
FOD2.5	Bottom	Three-band DI	(1145, 1344, 1137)	0.811
FOD2.5	Bottom	Three-band NDI	(1344, 1136, 351)	0.730
FOD2.5	Bottom	Three-band RI	(1136, 723, 721)	0.704

Note: Values in parentheses represent the selected three-band combination (i, j, k). All correlation coefficients are statistically significant at *p* < 0.05.

## Data Availability

The raw data supporting the conclusions of this article will be made available by the authors on reasonable request.
